# PHD1 Links Cell-Cycle Progression to Oxygen Sensing through Hydroxylation of the Centrosomal Protein Cep192

**DOI:** 10.1016/j.devcel.2013.06.014

**Published:** 2013-08-26

**Authors:** Sandra C. Moser, Dalila Bensaddek, Brian Ortmann, Jean-Francois Maure, Sharon Mudie, J. Julian Blow, Angus I. Lamond, Jason R. Swedlow, Sonia Rocha

**Affiliations:** 1 Centre for Gene Regulation and Expression, College of Life Sciences, University of Dundee, Dundee DD1 5EH, UK

## Abstract

PHD1 belongs to the family of prolyl-4-hydroxylases (PHDs) that is responsible for posttranslational modification of prolines on specific target proteins. Because PHD activity is sensitive to oxygen levels and certain byproducts of the tricarboxylic acid cycle, PHDs act as sensors of the cell’s metabolic state. Here, we identify PHD1 as a critical molecular link between oxygen sensing and cell-cycle control. We show that PHD1 function is required for centrosome duplication and maturation through modification of the critical centrosome component Cep192. Importantly, PHD1 is also required for primary cilia formation. Cep192 is hydroxylated by PHD1 on proline residue 1717. This hydroxylation is required for binding of the E3 ubiquitin ligase SCF^Skp2^, which ubiquitinates Cep192, targeting it for proteasomal degradation. By modulating Cep192 levels, PHD1 thereby affects the processes of centriole duplication and centrosome maturation and contributes to the regulation of cell-cycle progression.

## Highlights

• The prolyl-4-hydroxylase PHD1 is required for mitotic progression
• PHD1 hydroxylates centrosomal protein Cep192 in vitro and in vivo
• Hydroxylation of Cep192 at Pro1717 modulates Cep192 stability and function
• Hydroxylation of Cep192 is required for E3 ligase SCF^Skp2^ binding to Cep192

## Teaser

Cellular oxygen is sensed by a class of dioxygenase enzymes including the prolyl hydroxylase PHD1. Moser et al. find that PHD1 acts directly on the centrosome protein Cep192 to induce its SCF^Skp2^-dependent ubiquitination and degradation. PHD1 thus links oxygen sensing to the cell-cycle control of centrosome duplication and ciliogenesis.

## Introduction

One of the most important and fundamental processes in the cell is the precise duplication of genetic material during S phase and its segregation in mitosis. The execution of both of these processes without any mistakes is of crucial importance for the survival of the organism. Central to cell division is the alignment of mitotic chromosomes and the formation of the mitotic spindle. The mitotic spindle is organized by centrosomes, which are composed of centrioles and pericentriolar material (Avidor-Reiss and Gopalakrishnan, 2013). Centrioles are also important in the formation of cilia, which are structures required for sensing and movement (Nigg and Stearns, 2011). Defects in centrosomes and spindle formation give rise to a variety of human diseases (Noatynska et al., 2012), demonstrating the importance of these structures.

Cell division is also highly energy demanding and must be avoided when environmental conditions are not optimal. We anticipate that mechanisms exist to convey information about the metabolic state of the cell to the cell-cycle machinery, thereby preventing catastrophic events such as missegregation of genetic material or mitotic death.

One stimulus that has profound changes to the cell energy supply and metabolic state is hypoxia, or lowering of oxygen availability (Kenneth and Rocha, 2008). Hypoxia activates a transcriptional program mediated by the hypoxia-inducible factors (HIFs) (Rocha, 2007). In addition, hypoxia induces HIF-independent processes such as translational block and activation of additional transcription factors (Kenneth and Rocha, 2008; Rocha, 2007).

In humans, the three prolyl-4-hydroxylases PHD1–3 are well known for their role in regulating the stability of the transcription factor HIF in the response to oxygen levels (Fandrey et al., 2006). PHD activity relies on the availability of oxygen, iron, and 2-oxoglutarate (Jokilehto and Jaakkola, 2010). Upon hypoxia, the activity of PHDs decreases, which leads to the stabilization of the HIF proteins. This is due to the reduced affinity of the von Hippel Lindau protein (pVHL) ubiquitin E3 ligase toward the HIF proteins when proline hydroxylation is impaired. Recent evidence has shown that PHDs act not only as molecular oxygen sensors but also as sensor of errors in metabolism (Raimundo et al., 2011). Apart from HIF, two additional proteins, PKM2 (Luo et al., 2011) and HCLK2 (Xie et al., 2012), have been shown by mass spectrometry to be hydroxylated by PHD3. However, so far no additional targets for either PHD1 or PHD2 have been detected and validated.

There are several studies demonstrating that hypoxia alters the cell cycle (Kenneth and Rocha, 2008). For example, it is known that hypoxia induces a G1/S cell-cycle arrest via both HIF-dependent and -independent mechanisms (Culver et al., 2011; Hubbi et al., 2013). However, there has been no evidence clearly indicating a role for PHDs in the regulation of cell-cycle progression.

## Results

### Loss of PHD1 Inhibits Mitotic Progression

**Figure 1:**
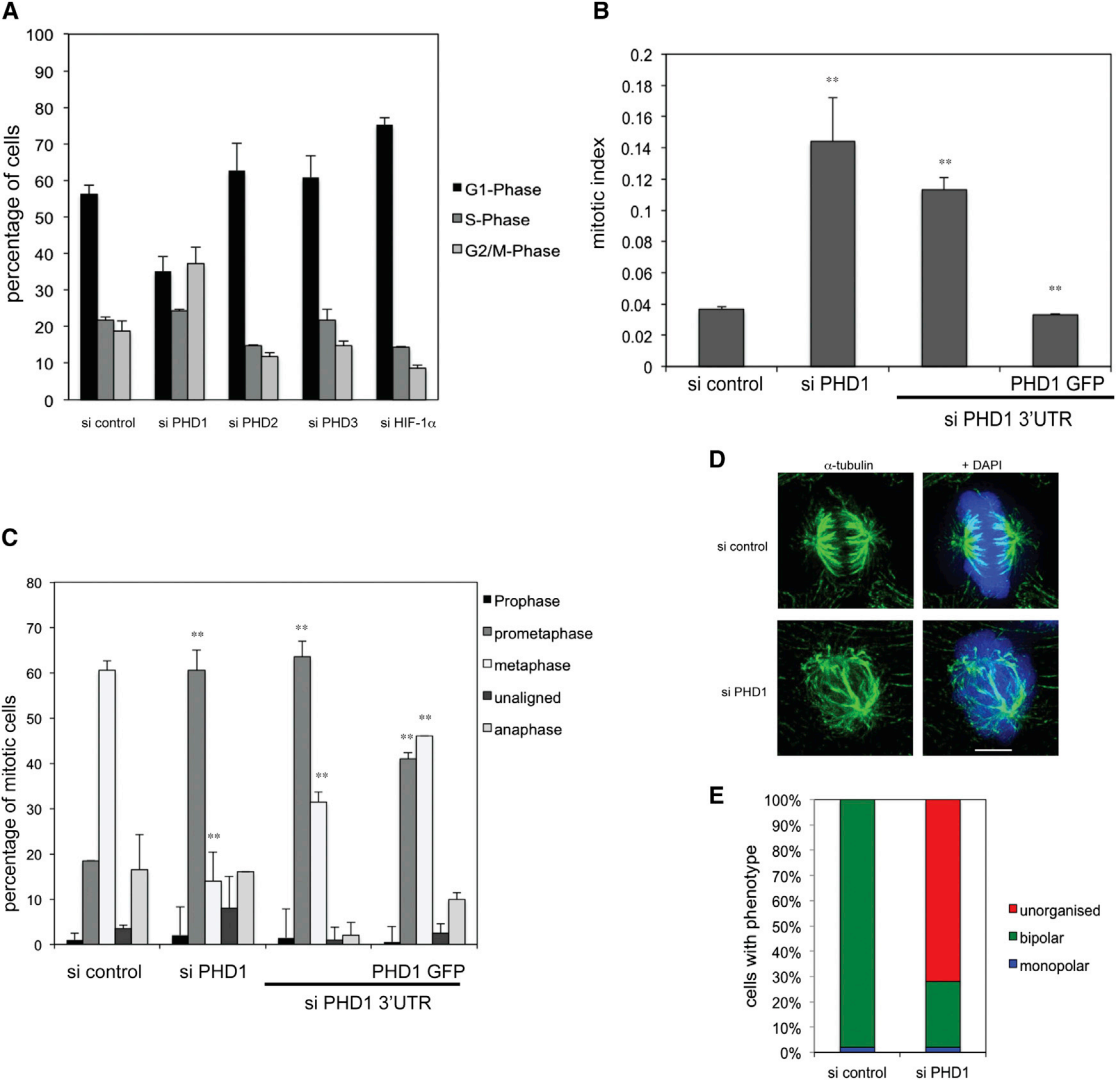
Loss of PHD1 Inhibits Mitotic Progression (A) HeLa cells were treated with the indicated siRNAs, and the cell-cycle profile was determined by flow cytometry. Values represent averages of two different experiments. Error bars indicate ± 1 SD. (B) Transfection of GFP-PHD1 rescues the mitotic arrest of HeLa cells treated with PHD1 siRNA. HeLa cells were transfected with the indicated siRNAs and plasmids and the mitotic index was determined. Values represent averages of three different experiments (n = 100; error bars indicate ± 1 SD; p values are significant according to the Student’s t test; ^∗∗^p < 0.01). (C) Transfection of GFP-PHD1 partially rescues the prometaphase arrest of HeLa cells treated with PHD1 siRNA. HeLa cells were transfected with the indicated siRNAs and plasmids and the mitotic distribution was determined. Values represent averages of two different experiments (n = 100; error bars indicate ± 1 SD; p values are significant according to the Student’s t test; ^∗∗^p < 0.01). (D) PHD1 depletion disrupts the mitotic spindle. Immunofluorescence images of mitotic cells treated with PHD1 siRNA and stained for α-tubulin (green) and DNA (blue). The scale bar represents 5 μm. (E) Quantification of spindle morphology phenotypes observed in PHD1-depleted cells. See also Figure S1.

To determine whether PHDs have a role in cell-cycle progression, we independently depleted each of the three isoforms PHD1–3 by siRNA in HeLa Kyoto cells (Figures S1A–S1D available online). Whereas depletion of either PHD2 or PHD3 caused little or no alteration of the cell-cycle profile, depletion of PHD1 increased the G2/M population of cells and decreased the G1 population (Figure 1A). In contrast, and as previously observed (Culver et al., 2011), HIF-1α depletion led to an increase of cells in G1 (Figure 1A). Further analysis revealed that PHD1 knockdown increased the mitotic index (Figure 1B). When the endogenous PHD1 protein was removed by targeting the 3′ UTR of its mRNA with siRNA, this effect could be reversed by the expression of PHD1 from a cDNA lacking the siRNA target site (Figure 1B). These data suggest that PHD1 is required for mitotic progression.

To characterize the PHD1 depletion phenotype, we determined whether cells would accumulate at a specific mitotic stage. Whereas most mitotic cells treated with an unspecific siRNA were in metaphase, cells depleted of PHD1 were delayed predominantly in prometaphase (Figure 1C). This prometaphase delay was partially rescued by the expression of exogenous PHD1 from a cDNA lacking the siRNA target site (Figure 1C). A similar effect was also observed in U2OS cells and in untransformed retinal pigment epithelial (RPE)1 cells, albeit to a lesser degree (Figures S1E and S1F). The difference between HeLa and U2OS cells is most likely due to differences in the transfection efficiency of these cells. To understand the basis for the prometaphase delay, we examined PHD1-depleted cells by immunofluorescence and observed disorganized mitotic spindles with poorly focused microtubules at the spindle pole (Figures 1D and 1E). These results suggest that PHD1 is required for the correct formation of the mitotic spindle and that the accumulation of cells in prometaphase following PHD1 depletion is likely to be a consequence of failure to form a functional spindle.

### PHD1 and Cep192 Regulate Centrosome Behavior

**Figure 2:**
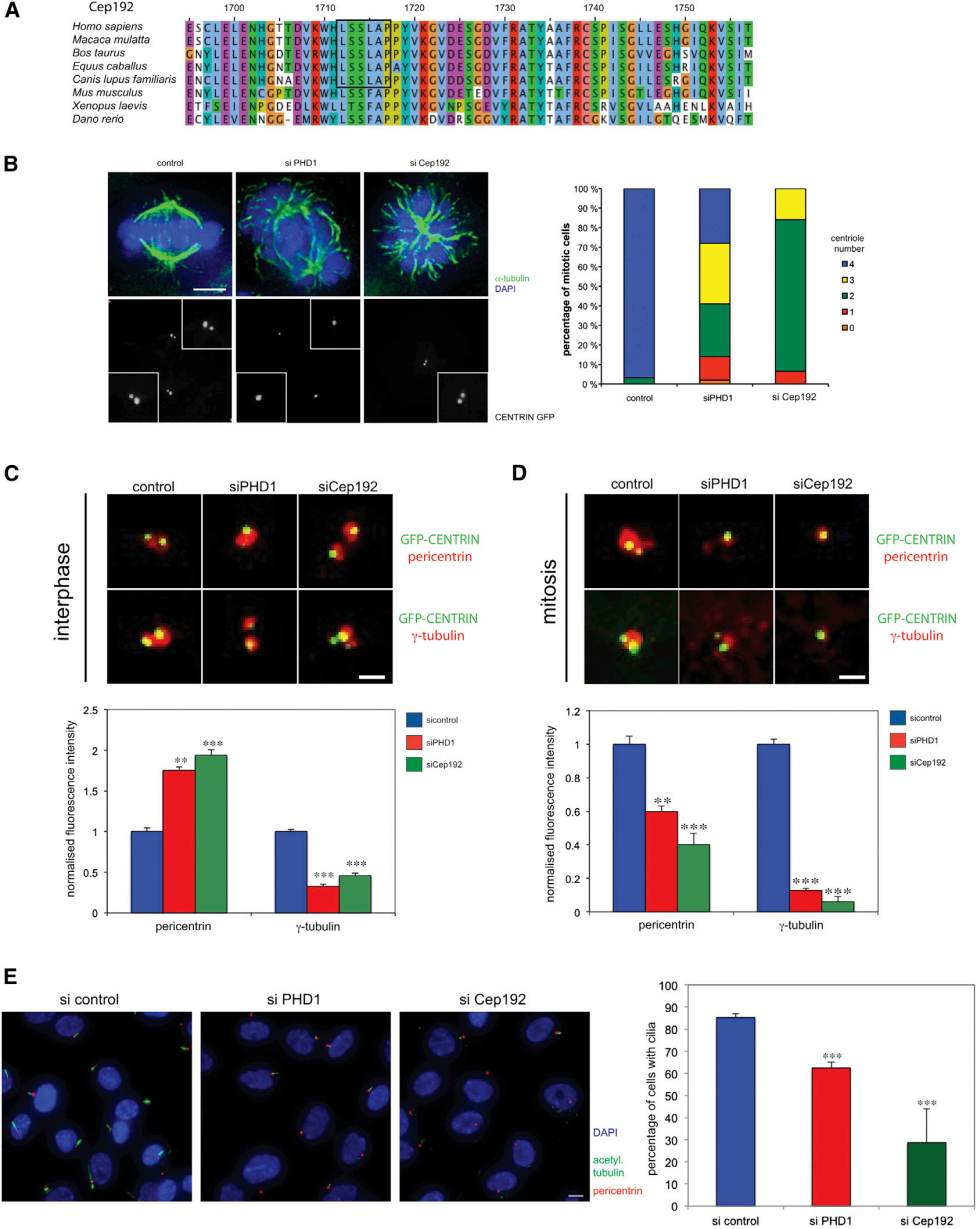
PHD1 and Cep192 Regulate Centrosome Behavior (A) Cep192 contains an LXXLAP motif. Protein sequence alignment of the centrosomal protein Cep192 from higher eukaryotes. Cep192 of higher mammals contains a potential PHD hydroxylation motif (black box). (B) PHD1 and Cep192 are required for centriole duplication. HeLa cells were treated with PHD1 or Cep192 siRNA. Immunofluorescence pictures (upper left panels) show mitotic cells treated with siRNA and stained for α-tubulin (green) and DNA (blue). Lower left panels display centrioles of mitotic cells as marked by GFP-centrin. Insets focus on the centrioles. The scale bar represents 5 μm. The centriole number per mitotic cell was determined for each treatment (right panel) (n = 100). (C) PHD1 affects localization of centrosomal components during interphase. Cells expressing GFP-centrin (green) were treated with PHD1 and Cep192 siRNA and stained with antibodies against pericentrin (red, top panels) and γ-tubulin (red, bottom top panels). The scale bar represents 1 μm. The normalized fluorescence intensity of pericentrin and γ-tubulin was determined (bottom panel) (n = 20; error bars indicate ± 1 SEM; p values are significant according to the Student’s t test; ^∗∗^p < 0.01, ^∗∗∗^p < 0.0001). (D) PHD1 affects centrosome maturation in mitosis. Cells expressing GFP-centrin (green) were treated with PHD1 and Cep192 siRNA and stained as in (C). The scale bar represents 1 μm. The normalized fluorescence intensity of pericentrin and γ-tubulin was determined (bottom panel) (n = 20; error bars indicate ± 1 SEM; p values are significant according to the Student’s t test; ^∗∗^p < 0.01, ^∗∗∗^p < 0.0001). (E) PHD1 is required for the formation of primary cilia. RPE cells were depleted of PHD1 or Cep192 and stained for acetylated tubulin (green) and pericentrin (red) to mark primary cilia and DNA (blue). The scale bar represents 10 μm. The percentage of cells in which primary cilia were formed by the centrosome as marked by pericentrin was determined. Bars represent the average of two different experiments (n = 100; error bars indicate ± 1 SD; p values are significant according to the Student’s t test; ^∗∗∗^p < 0.0001). See also Figure S2.

Most of the known PHD substrate proteins have an LXXLAP motif in which the proline is modified by hydroxylation. To identify potential mitotic spindle PHD1 substrates, we scanned the human proteome for proteins containing this motif and having a known function in spindle formation. The centrosomal protein Cep192 matched both criteria (Figure 2A). Cep192 is a component of the pericentriolar material of centrosomes. It was first identified as SPD-2 in *Caenorhabditis elegans*, where it is known to be required for centriole duplication and centrosome maturation (Kemp et al., 2004; Pelletier et al., 2004). These functions are shared with its human counterpart (Gomez-Ferreria et al., 2007; Zhu et al., 2008). Mammals such as primates, horse, dog, and cow possess a Cep192 protein with a conserved PHD consensus motif (Figure 2A). However, the consensus motif is not found in rodents, such as mouse or lower vertebrates.

To address whether Cep192 is a potential target of PHD1, we first tested whether PHD1 is required for centriole duplication. We depleted either Cep192 or PHD1 in HeLa cells using siRNA and determined centriole numbers in mitotic cells. Whereas most mitotic cells treated with an unspecific siRNA displayed four centrioles, the majority of cells with reduced Cep192 levels only possessed two centrioles (Figure 2B). This phenotype was also observed using a separate siRNA oligonucleotide targeting Cep192 (Figure S2A). PHD1 depletion also resulted in reduced centriole numbers, with most cells possessing two or three centrioles per mitotic cell (Figure 2B). This suggests that, like Cep192, PHD1 is required for centriole duplication.

Cep192 is also required for centrosome maturation during mitosis, which involves an increase of centrosomal components such as pericentrin and γ-tubulin (Kemp et al., 2004; Zhu et al., 2008). We therefore examined the effects of PHD1 depletion on the targeting of pericentrin and γ-tubulin to the centrosome in interphase and mitosis. In interphase, cells depleted with siRNA against either PHD1 or Cep192 had less γ-tubulin but more pericentrin at the centrosome (Figure 2C; Figure S2A). In mitosis, both γ-tubulin and pericentrin failed to efficiently target the centrosome in the absence of either PHD1 or Cep192 (Figure 2D; Figure S2B). Cep192 is also required for the formation of primary cilia, microtubule-based antenna-like structures that emanate from the surface of virtually all cells in the body and mediate many signaling pathways and mechanochemical reactions (Gönczy, 2012; Nigg and Raff, 2009). Similar to the loss of Cep192, depletion of PHD1 results in a decrease of primary cilia (Figure 2E).

Taken together, these results indicate that both Cep192 and PHD1 are required for proper centrosome formation and function in a cell-cycle-dependent manner, affecting duplication, maturation, and primary cilia formation.

### Cep192 Is Hydroxylated by PHD1

**Figure 3:**
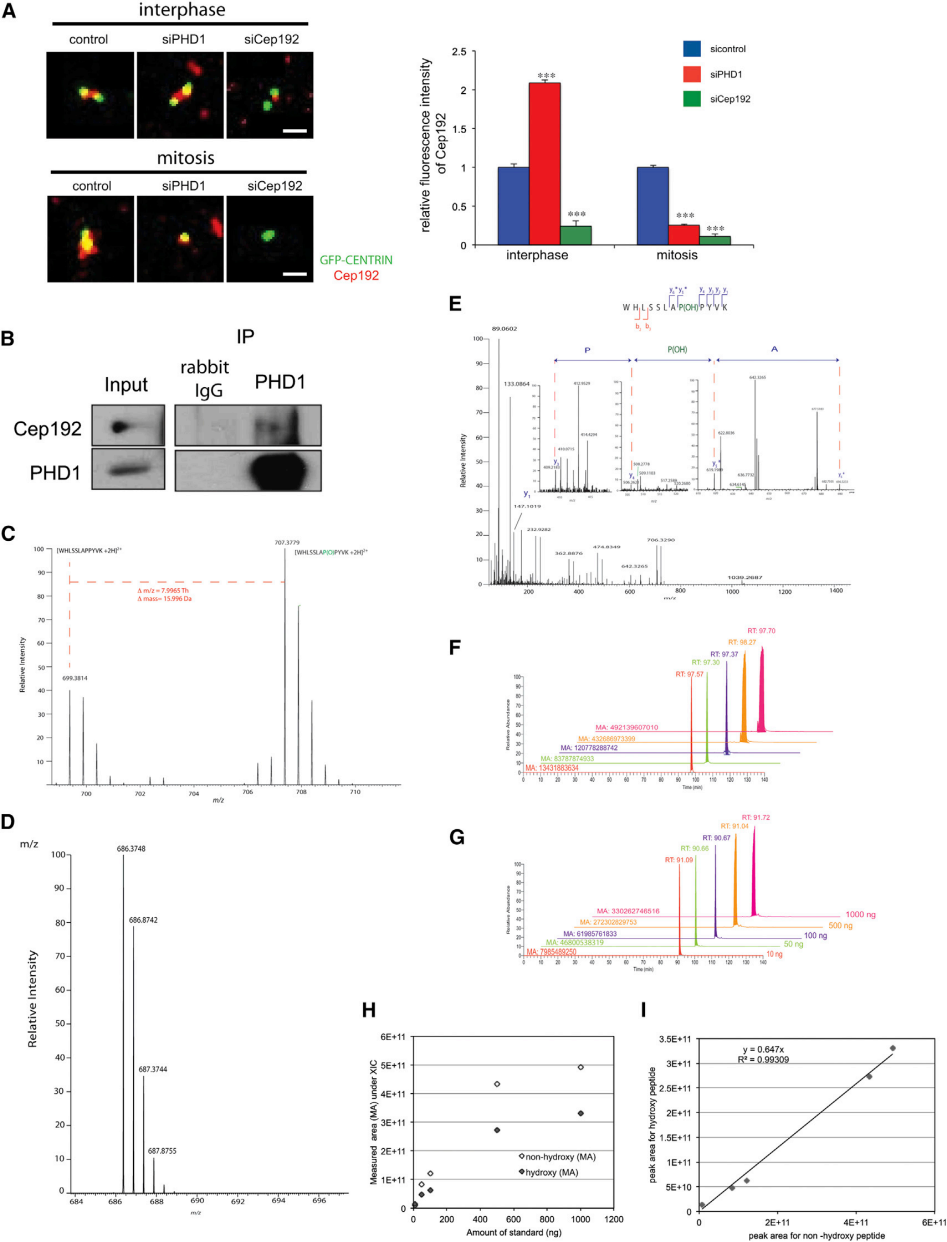
Cep192 Is a Hydroxylation Target of PHD1 (A) PHD1 is required for the centrosomal localization of Cep192. HeLa cells expressing GFP-centrin (green) were depleted of PHD1 or Cep192 and stained for Cep192 (red) in interphase (left panel, upper) and mitosis (left panel, lower). The scale bars represent 1 μm. The relative fluorescence intensity of Cep192 signal in interphase and mitosis was determined (right panel) (n = 20; error bars indicate ± 1 SEM; p values are significant according to the Student’s t test; ^∗∗∗^p < 0.0001). See also Figure S2. (B) Cep192 interacts with PHD1. PHD1 was immunoprecipitated from cells and blotted for Cep192. See also Figure S3. (C) Electrospray-MS spectrum of the product of in vitro hydroxylation of the synthetic peptide WHLSSLAPPYVK showing an *m/z* increment of 5.333 Th (the mass increment of 15.999 Da) corresponding to proline hydroxylation of the triply charged ion at *m/z* 466.5894 Th and the formation of the ion 471.9224 Th (the mass of the hydroxylated peptide). (D) Absence of an *m/z* increment of 5.333 Th (or mass increment of 15.999 Da) of the triply charged ion, indicating that the peptide WHLSSLAAPYVK is not in vitro hydroxylated. (E) Cep192 is hydroxylated on proline 1717. Product ion spectrum of the endogenous doubly charged ion at *m/z* 707.3797 Th corresponding to the hydroxylated peptide WHLSSLAP(OH)PYVK showing b and y fragment ions typical for higher-energy C trap dissociation fragmentation, which allowed the identification of the peptide sequence. Asterisks denote the fragment ions incorporating the mass increment of 15.999 Da, corresponding to proline hydroxylation. See also Figure S4. (F and G) Titration of nonhydroxylated (F) and hydroxylated standards (G). Diagrams show extracted-ion chromatograms, retention times, and measured areas for the standards injected in different amounts. (H) Diagram correlating the MAs of signals for nonhydroxylated and hydroxylated standards to the amounts of material analyzed. (I) Correlation of the MA of nonhydroxylated standard and hydroxylated standard. See also Figures S3 and S4.

We next explored whether Cep192 localization depends on PHD1, and found that PHD1 depletion by siRNA resulted in an increase of Cep192 at the centrosome during interphase and a failure to accumulate Cep192 at the centrosome during mitosis (Figure 3A). To determine whether PHD1 is associated with complexes containing Cep192, we isolated PHD1 by immunoprecipitation and observed the copurification of Cep192 (Figure 3B). PHD1 also colocalized with Cep192 during mitosis, as judged by fluorescence microscopy (Figure S3). Although PHD1 is mainly observed in the nucleus (Metzen et al., 2003), biochemical analysis demonstrated that, like Cep192, a proportion of PHD1 also localizes to the cytoplasm (Figure S3B). Based on these data, we next tested whether PHD1 hydroxylates Cep192. In vitro recombinant PHD1 efficiently hydroxylated a Cep192 peptide containing the motif LXXLAP (Figure 3C) but not a peptide in which the putative modified proline was mutated to an alanine (Figure 3D). We next analyzed immunoprecipitates of Cep192-GFP isolated from HeLa Kyoto cells that stably express Cep192-GFP at endogenous levels by nano-LC (liquid chromatography) and targeted tandem MS scanning on a high-resolution mass spectrometer. In these immunoprecipitates, we identified a peptide ion of Cep192 that was hydroxylated at proline 1717 in vivo (Figure 3E; Figure S4). To determine the stoichiometry of Cep192 hydroxylation, a quantification method was developed in which the level of in-vivo-hydroxylated Cep192 was compared with known amounts of synthetic hydroxylated and nonhydroxylated standards (see Experimental Procedures). From these data, we calculate that ∼10% of Cep192 is hydroxylated at Pro1717 in cells growing in asynchronous culture (Figures 3F and 3G).

### Hydroxylation of Cep192 at Pro1717 Is Required for Cep192 Function

**Figure 4:**
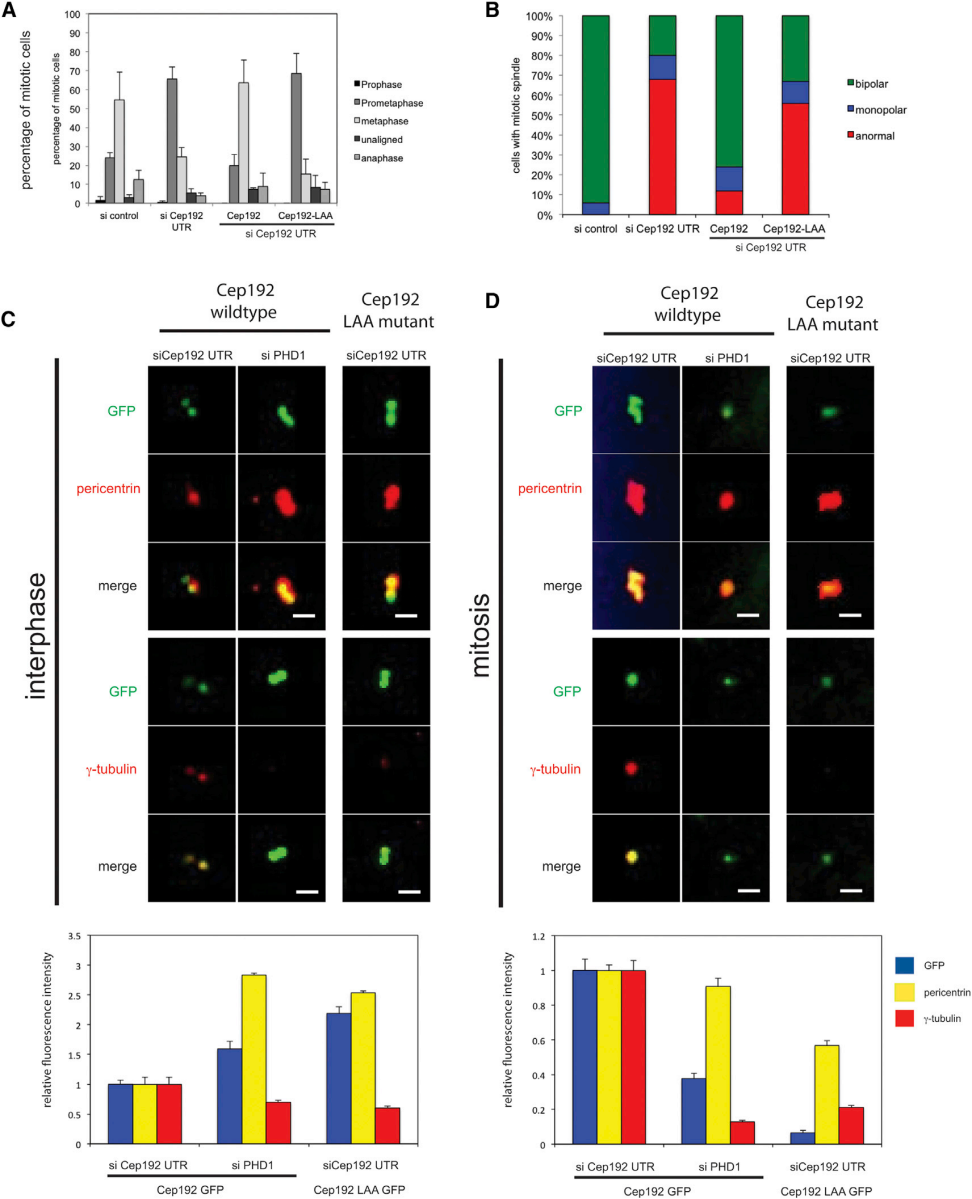
Nonhydroxylatable Cep192 Interferes with Centrosome Function (A) Cells expressing nonhydroxylatable Cep192 accumulate in prometaphase. HeLa cells stably expressing the indicated constructs were transfected with the indicated siRNAs and the mitotic distribution was determined. Values represent averages of three different experiments (n = 100; error bars indicate ± 1 SD). (B) Nonhydroxylatable Cep192 disrupts the mitotic spindle. Quantification of spindle morphology phenotypes observed in cells stably expressing the indicated constructs and depleted with the indicated siRNAs. (C) Cep192 mutated on proline 1717 accumulates at the centrosome in interphase. HeLa cells stably expressing wild-type or the LAA mutant of Cep192-GFP (green) were treated with the indicated siRNAs and probed for pericentrin (red) (top panels). The scale bars represent 1 μm. The relative fluorescence intensity of Cep192-GFP, pericentrin, and γ-tubulin in interphase cells was determined (bottom panel) (n = 20; error bars indicate ± 1 SEM; p values are significant according to the Student’s t test; ^∗∗^p < 0.01, ^∗∗∗^p < 0.0001). (D) The LAA mutant of Cep192 does not accumulate at the centrosome in mitosis. Panels as in (C). The relative fluorescence intensity of Cep192-GFP, pericentrin, and γ-tubulin in mitotic cells was determined (bottom panel) (n = 20; error bars indicate ± 1 SEM; p values are significant according to the Student’s t test; ^∗∗^p < 0.01, ^∗∗∗^p < 0.0001).

To determine the functional significance of the proline hydroxylation of Cep192, we mutated proline 1717 to alanine and analyzed the effect of the mutation on Cep192 function. Consistent with our data described above, depletion of Cep192 using siRNA increased the fraction of cells in prometaphase. This could be rescued when wild-type Cep192 was expressed from a cDNA lacking the siRNA target site. However, cells expressing the single-site mutation Cep192^P1717A^ accumulated in prometaphase in a similar manner to cells depleted of the WT protein (Figure 4A). Mitotic spindles in Cep192-depleted cells were severely disorganized, and this phenotype was rescued when cells were transfected with siRNA-resistant WT Cep192 but not in cells transfected with Cep192^P1717A^ (Figure 4B).

We next examined the effects of mutating Pro1717 in terms of Cep192 localization in cells during the cell cycle. When compared with WT Cep192, expression of Cep192^P1717A^ caused an increase of Cep192 present on centrosomes in interphase (Figure 4C) and a decrease of Cep192 on centrosomes in mitosis (Figure 4D) in a manner analogous to the effect caused by PHD1 knockdown (Figures 4C and 4D). Furthermore, Cep192^P1717A^ expression resulted in an increase of pericentrin and a decrease of γ-tubulin in interphase (Figure 4C), whereas in mitosis, levels of pericentrin and γ-tubulin on centrosomes were decreased (Figure 4D). This is also consistent with the phenotypes we observed in cells depleted of PHD1 (Figure 4D). These results indicate that PHD1 hydroxylates Cep192 on proline 1717 and that PHD1-mediated hydroxylation of proline 1717 is important for the centrosomal localization and function of Cep192 throughout the cell cycle.

**Figure 5:**
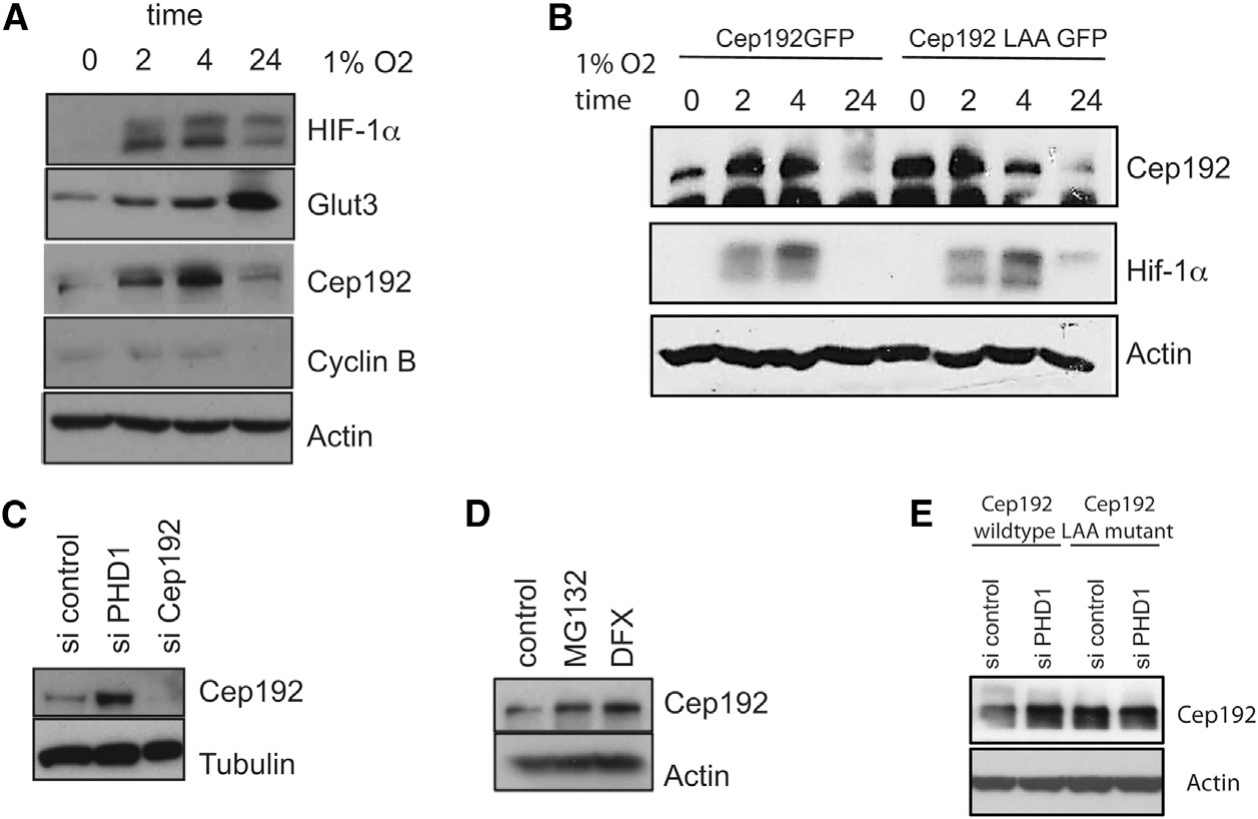
Changes in PHD1 Activity Alter Cep192 Protein Levels (A) Hypoxia leads to the increase of Cep192 levels. U2OS cells were exposed to 1% O_2_ for the indicated time points. Whole-cell lysates were blotted for the indicated proteins. (B) Low-oxygen tension increases Cep192-GFP levels but not Cep192-LAA-GFP levels. HeLa Kyoto cells stably expressing Cep192-GFP and Cep192-LAA-GFP were exposed to 1% O_2_ for the indicated time points. Whole-cell lysates were blotted for the indicated proteins. (C) Inactivation of PHD1 increases Cep192 levels. Cells were treated with PHD1 siRNA and lysates were blotted for the indicated proteins. (D) Cells were treated with DFX or MG132 and lysates were blotted for the indicated proteins. (E) Inactivation of PHD1 increases Cep192-GFP levels but not that of the Cep192-LAA-GFP mutants. Cells were treated with PHD1 siRNA and lysates were blotted for the indicated proteins. See also Figures S5 and S6.

Exposure of cells to hypoxia inhibits PHDs, which then stabilizes the HIF proteins (Figure 5A). If Cep192 is a substrate of PHD1, we reasoned that Cep192 hydroxylation and stability would also be sensitive to oxygen tension. Indeed, reducing oxygen levels caused an increase in Cep192 levels that paralleled the well-established stabilization of HIF-1α (Figures 5A and 5B). In contrast, Cep192^P1717A^ levels did not increase in response to hypoxia (Figure 5B). Likewise, either depletion of the PHD1 protein by siRNA or treatment of cells with the hypoxia-mimetic desferrioxamine (DFX) led to an increase of Cep192 protein levels (Figures 5C and 5D). Finally, whereas Cep192^P1717A^ levels were higher than WT Cep192 (Figure 5E), depletion of PHD1 by siRNA treatment did not lead to any further increase in the level of Cep192^P1717A^. On the other hand, wild-type Cep192 levels increased when PHD1 was depleted with siRNA (Figure 5E).

Given the responsiveness of Cep192 to hypoxia, DFX, and PHD1 depletion, it was possible that these effects were mediated by HIF. Therefore, we next investigated whether Cep192 is a transcriptional target of HIF. To test this, we first analyzed Cep192 mRNA levels upon PHD1 depletion by siRNA (Figure S5A). PHD1 knockdown resulted in a slight decrease of Cep192 mRNA, suggesting that Cep192 is not a transcriptional target of HIF, as there was no increase in Cep192 mRNA in the absence of PHD1 (Figure S5A). In addition, Cep192 mRNA levels were not changed upon HIF isoform depletion (Figure S5B), and Cep192 protein levels remained unchanged in the absence of HIF-1α (Figure S5C). Taken together, these data indicate that Cep192 is not a target of regulation by the HIF transcription factors.

Although Cep192 protein levels increased following either hypoxia or DFX addition, we did not know whether this was reflected in a concomitant change in the level of Cep192 accumulating at the centrosome. We therefore tested this by fluorescence microscopy following exposure of cells to hypoxia. This showed that the amount of Cep192 at the centrosome was dramatically decreased, as was pericentrin (Figures S6A and S6B). In contrast, Cep192 levels at the centrosome increased upon the addition of the iron chelator DFX (Figures S6C and S6D). These results suggest that hypoxia affects centrosome assembly and structure not only through PHDs but also through additional mechanisms that prevent proper Cep192 localization.

### Hydroxylation of Cep192 Is Required for Ubiquitination and SCF^Skp2^ Binding

**Figure 6:**
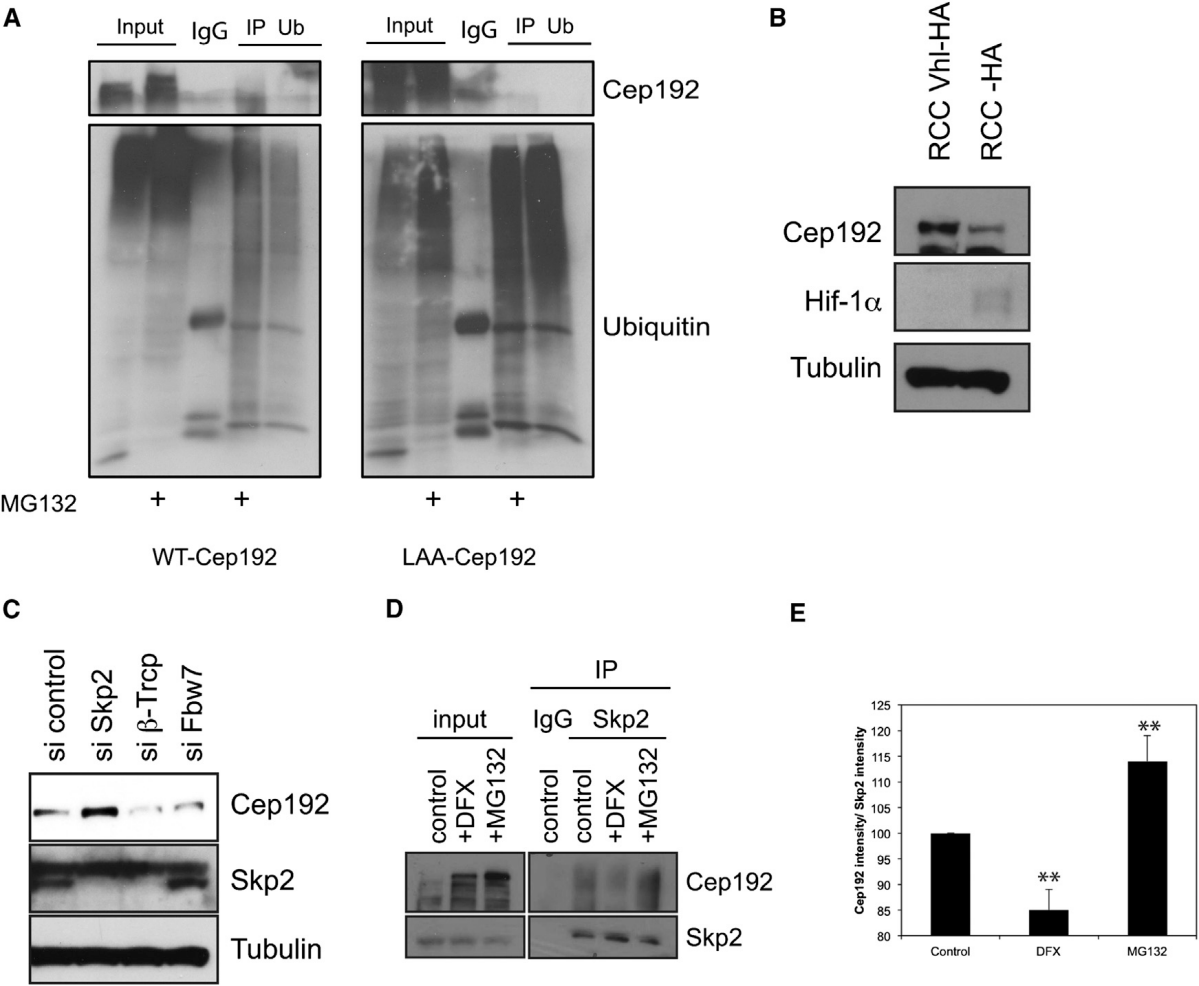
Hydroxylation of Cep192 Targets the SCF^Skp2^ Complex (A) Cep192 is ubiquitinated. HeLa cells expressing Cep192-GFP or Cep192-LAA-GFP were treated with MG132 for 3 hr, and then lysates were immunoprecipitated with control IgG or with anti-ubiquitin antibody and immunoprecipitates were blotted for Cep192 (upper panels) or ubiquitin (lower panels). (B) Cep192 stability is not dependent on pVHL. Whole-cell lysates of renal cell carcinoma (RCC) cells and RCC cells reconstituted with HA-pVHL were blotted for the indicated proteins. (C) Cep192 destabilization is dependent on Skp2. HeLa cells were treated with the indicated siRNAs and whole-cell extracts were blotted with the indicated antibodies. (D) Cep192 interaction with Skp2 depends on hydroxylation. Cells were treated with MG132 or DFX for 3 hr, and then lysates were immunoprecipitated with control IgG or with anti-Skp2 antibody and immunoprecipitates were blotted for Cep192. (E) Quantification of Cep192-Skp2 interaction. Values represent averages of four different experiments. Error bars represent SD. p values are significant according to the Student’s t test; ^∗∗^p < 0.01.

Proline hydroxylation of HIF-α proteins is necessary for their ubiquitination and subsequent proteasomal degradation. To test whether a similar mechanism is regulating Cep192 levels, we first addressed whether Cep192 is a target of the proteasome. When the proteasome was inhibited by the addition of MG132, Cep192 levels increased slightly (Figure 5D). To determine whether Cep192 was ubiquitinated and whether the hydroxylation was required for this process, we immunoprecipitated ubiquitinated proteins from cells plus or minus prior exposure to MG132, and then blotted the immunoprecipitates for Cep192. Control analysis demonstrated that ubiquitin was immunoprecipitated from both cell types (Figure 6A, lower panels). Whereas wild-type Cep192 protein isolated from cell lysates was ubiquitinated, nonhydroxylatable Cep192 mutant protein was not, as we could not detect Cep192 in the immunoprecipitates from these cells (Figure 6A, top panels).

PHD-mediated hydroxylation of HIF results in pVHL-dependent ubiquitination and degradation (Fandrey et al., 2006). However, in contrast to HIF-α proteins, the degradation of Cep192 was independent of pVHL, as Cep192 levels were not elevated in pVHL-deficient cells (Figure 6B). Other E3 ligases known to be active during interphase and present at the centrosome are Brca1 and the SCF (Skp, Cullin, F box) complex. Brca1 depletion had no effect on Cep192 levels (data not shown). Although Cep192 has potential binding sites for the SCF F box proteins β-Trcp and Fbw7, when these enzymes were depleted by siRNA, we could not detect a change in Cep192 levels (Figure 6C). However, depletion of the F box protein Skp2 resulted in increased Cep192 levels (Figure 6C), suggesting that Skp2 might regulate Cep192 in a PHD1-dependent manner. We found that Skp2 was coimmunoprecipitated with Cep192 and that this interaction was decreased after DFX addition (Figures 6D and 6E). These results suggest that the hydroxylation of Cep192 by PHD1 is required for the ubiquitination of Cep192 by Skp2.

### Overexpression of Cep192 Interferes with Centriolar Duplication

**Figure 7:**
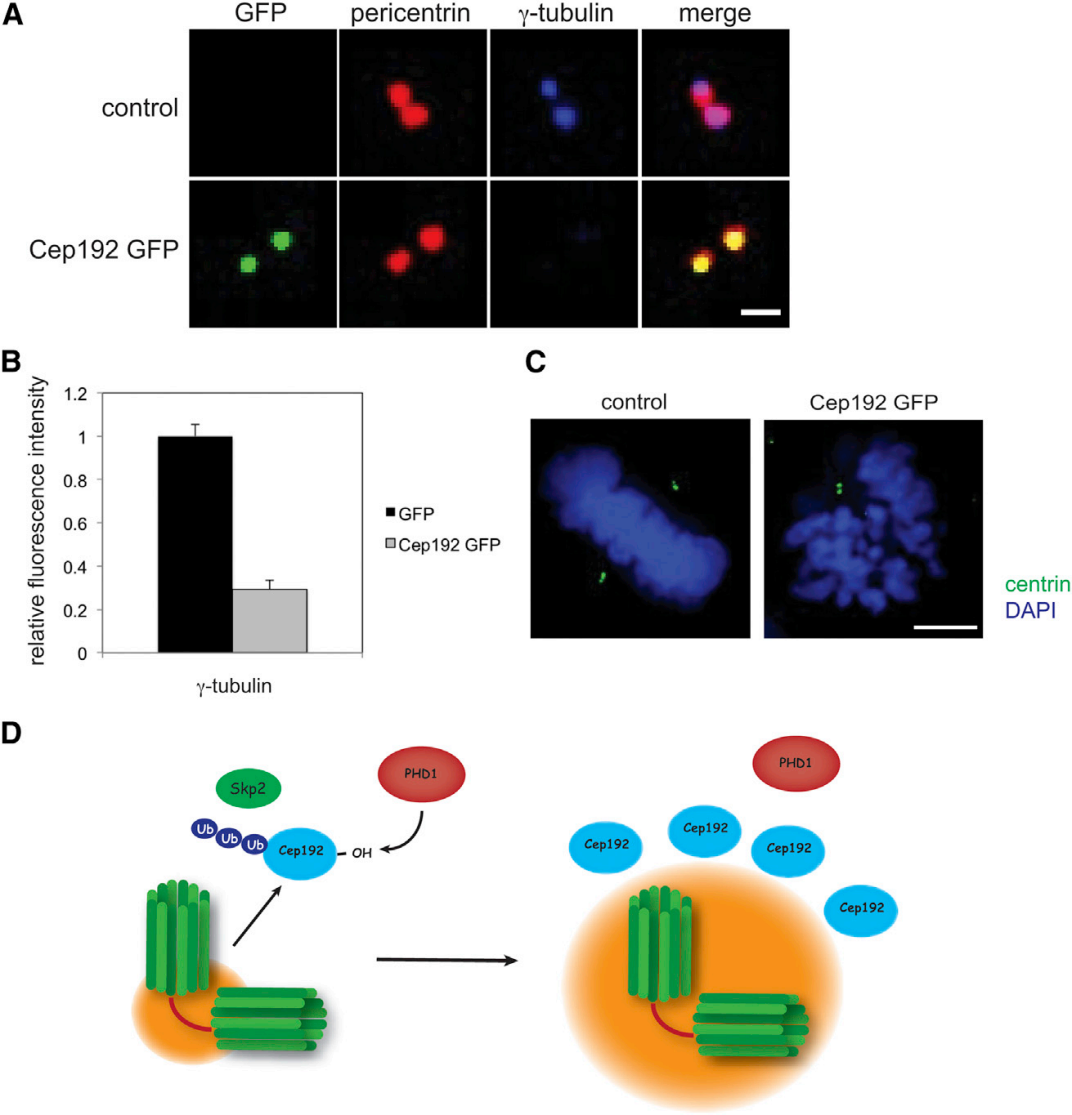
Cep192 Overexpression Phenotype Overlaps with PHD1 Depletion (A) Cep192 overexpression interferes with centrosomal recruitment of γ-tubulin. HeLa cells were transfected with Cep192-GFP (green) and stained for pericentrin (red) and γ-tubulin (blue). The scale bar represents 1 μm. (B) Quantification of the relative fluorescence intensity of γ-tubulin. Error bars represent SD. (C) Overexpression of Cep192 interferes with centriolar duplication. HeLa cells transfected with Cep192-GFP were stained for centrin (green) and DNA (blue). Phenotype was observed in n = 8/20. The scale bar represents 5 μm. (D) Schematic of the proposed model for PHD1 regulation of Cep192.

Our results so far suggested that Cep192 is required to ensure proper centrosome function. However, PHD1 depletion results in higher levels of Cep192, but with similar effects on centrosomal function to Cep192 depletion. Therefore, we tested whether overexpression of Cep192 would have similar effects on the centrosome as PHD1 or Cep192 depletion. When we transfected HeLa cells with WT Cep192 cDNA, we noticed that γ-tubulin levels at the centrosome decreased (Figures 7A and 7B), whereas transfected mitotic cells showed reduced numbers of centrioles (Figure 7C). These results indicate that overexpression of WT Cep192 has serious consequences, possibly because excess amounts of Cep192 can interfere with centriolar duplication by disrupting the recruitment of γ-tubulin by the centrosome.

## Discussion

In this study, we have uncovered a regulatory mechanism involved in the control of cell-cycle progression that is mediated by controlling the expression levels of the centrosome protein Cep192 through targeted proline hydroxylation by PHD1. Our results demonstrate that the levels of Cep192 must be tightly regulated to ensure efficient centriole duplication, centrosome maturation, and spindle assembly. We show that Cep192 is ubiquitinated and targeted for degradation by the proteasome through hydroxylation of Pro1717 in Cep192 by PHD1. Mutation of Cep192 at proline 1717 to prevent hydroxylation results in abnormal centrosomal accumulation of Cep192 in interphase and a failure to form a functional mitotic spindle. The data therefore provide a link whereby PHD1 allows readout of the metabolic state in the cell to prevent inappropriate cell division.

The importance of strictly controlling Cep192 levels through the cell cycle is consistent with previous results (Gomez-Ferreria et al., 2007). Low levels of Cep192 are present at the centrosome in interphase and are necessary for centriole duplication. Overexpression of Cep192 causes a decrease of γ-tubulin on the centrosome and interferes with centriole duplication. Surprisingly, Cep192 knockdown has similar effects and causes a loss of γ-tubulin (Zhu et al., 2008) at the centrosome and a failure of centriole duplication. We conclude that too much or too little Cep192 on the centrosome is detrimental to the cell. This fact might also be the reason for our inability to rescue the centriole duplication phenotype of Cep192, as the level of expression of exogenous Cep192 cannot be as stringently regulated.

The high level of regulation of Cep192 protein levels might be necessary, as Cep192 creates a landing platform for factors necessary for centriolar duplication, but when excess levels of Cep192 are present, this interferes with the recruitment of additional factors and hence inhibits centriolar duplication. Similarly, excess Cep192 might interfere with centrosome maturation, although further studies are necessary to address the mechanism involved. For example, it will be interesting in the future to investigate what domains are involved in the distinct functions of Cep192, whether additional posttranslational modifications may also govern Cep192 levels and function, and how Cep192 is transcriptionally regulated. Nonetheless, the present data showing the control of Cep192 stability by PHD1 activity represent an example where centrosome biogenesis is controlled by a mechanism in which metabolic status is used to regulate protein stability.

Coupling the regulation of Cep192 to oxygen availability provides an elegant way to stop cell-cycle progression in response to changes in PHD activity, which can arise due either to oxygen deprivation or metabolic perturbation. Major cell-cycle processes, including DNA replication and mitosis, are energy-consuming events best avoided when cell-growth conditions are suboptimal. In addition to responding to oxygen levels, the activity of PHDs has also been shown to be sensitive to lack of iron (Fandrey et al., 2006), certain products of glycolysis (Fandrey et al., 2006), and, more recently, amino acids (Durán et al., 2012). Therefore, PHDs provide a mechanism to link the detection of environmental conditions to the regulation of cell-cycle progression. Germline mutations in enzymes involved in glycolysis, which also cause misregulation of PHDs, result in tumor formation (Raimundo et al., 2011). Although PHD1 has not previously been linked directly to mechanisms involved in cell transformation and cancer, PHD1 misregulation has been detected in forms of human breast, ovarian, and intestinal cancer (Jokilehto and Jaakkola, 2010). Formation of primary cilia, which we show here, is sensitive to PHD1 depletion, is also impaired in breast cancer cells (Yuan et al., 2010). Our data here, showing the involvement of PHD1 in modulating the levels of the Cep192 protein, which is important for the control of cell division, therefore provide a potential mechanistic link between loss of PHD1 function and disease.

As well as detecting an example of regulation acting via posttranslational proline hydroxylation, this analysis provides a direct measurement of the stoichiometry of proline hydroxylation at a regulated site. Thus, in an asynchronous cell population, we detected ∼10% of Cep192 hydroxylated at residue Pro1717. Using the method developed in this study, it will now be possible to also measure the stoichiometry of proline hydroxylation for other proteins regulated by similar mechanisms. This is not yet known for either the PHD-targeted HIF-α proteins or for the recently discovered PHD3 targets PKM2 and HCKL2. It will be interesting therefore to determine whether they are hydroxylated at a similar or higher level than Cep192 and, if so, under what conditions.

## Experimental Procedures

### Plasmid Constructs

The coding sequence for Cep192 was PCR cloned into pcDNA5/FRT (Invitrogen) and pEGFP-N1 (Clontech). A pEGFP-N1 construct for PHD1 was obtained from Addgene. PHD1 was PCR cloned into pcDNA5/FRT (Invitrogen). The LAA mutant of Cep192 was constructed by site-directed mutagenesis using a QuikChange Kit (Stratagene).

### Cilia Formation

hTERT-RPE1 cells were grown to 100% confluency. To induce primary cilia formation, the medium was then replaced with 0.25% FCS containing medium for 48 hr.

### siRNA Transfections

HeLa Kyoto and U2OS cells were transfected with Lipofectamine RNAiMax (Invitrogen) or Interferin (Peqlab). hTERT-RPE1 cells were transfected by electroporation using the Neon transfection system (Invitrogen).

#### siRNA Sequences

Control, 5′-CAGUCGCGUUUGCGACUGG-3′ (Culver et al., 2010); PHD1, 5′-GACUAUAUCGUGCCCUGCAUG-3′ (Culver et al., 2010); PHD1_UTR, 5′-GGACCAAGGAGGAGAGAG-3′; Cep192, 5′-GGAAGACAUUUUCAUCUCU-3′; Cep192_UTR, 5′-ACUGAAGACUCGACUGAAA-3′; Skp2, 5′-ACUCAAGUCCAGCCAUAAG-3′ (Barré and Perkins, 2010); Fbw7, 5′-ACAGGACAGUGUUUACAAA-3′ (Arabi et al., 2012); β-TrCP, 5′-GUGGAAUUUGUGGAACAUC-3′ (Kenneth et al., 2010); HIF-1α, 5′-CUGAUGACCAGCAACUUGA-3′ (Culver et al., 2010); PHD2, 5′-GACGAAAGCCAUGGUUGCUUG-3′ (Culver et al., 2010); PHD3, 5′-GUCUAAGGCAAUGGUGGCUUG-3′ (Culver et al., 2010).

### Immunoprecipitation for Mass Spectrometry

HeLa Kyoto cells expressing a human Cep192-GFP construct (gift of Tony Hyman; MCB_0002229/T no. 158) were treated with 40 μM MG132 for 4 hr. Cells were then washed twice with PBS and lysed in 20 mM Tris (pH 7.5), 150 mM NaCl, 0.5% Triton X-100, and EDTA-free complete protease inhibitor mix (Roche). Cell lysates were then incubated with 200 μl magnetic GFP-Trap beads (ChromoTek) for 2 hr. The beads were washed three times in lysis buffer and twice with PBS. The beads were resuspended in 20 μl SDS sample buffer. After the removal of the magnetic beads, samples were run on a Coomassie gel and stained with Instant blue (Invitrogen). The band corresponding to the molecular weight of Cep192-GFP was excised with a scalpel.

### In-Gel Digestion

The gel pieces were washed in 100 mM triethylammonium bicarbonate:acetonitrile (50:50; v/v) to remove the Coomassie stain. The proteins were reduced with DTT (50 mM) in triethylammonium bicarbonate at 65°C and alkylated using iodoacetamide (50 mM) at room temperature in the dark. The gel pieces were washed with acetonitrile to remove excess reagent and dried in vacuo prior to protein digestion. To each gel band, 300 ng of tosyl phenylalanyl chloromethyl ketone-treated porcine trypsin (Roche) was added and incubated overnight at 37°C. After overnight incubation, the resulting peptides were extracted using acetonitrile:water:formic acid (50:49.9:0.1) three times and dried under vacuum. The dried tryptic peptides were dissolved in 5% formic acid and analyzed by LC-MS/MS.

### Quantification of the Stoichiometry of Hydroxylation

Hydroxylated and nonhydroxylated peptides were injected on a reverse-phase column in increasing amounts (10, 50, 100, 500, and 1,000 ng) and analyzed using a short gradient.

We attempted to correlate the peak intensities and the areas under the peak to the amount of peptide analyzed. We analyzed each standard peptide on its own to correlate the peak properties to the amount of peptide used and then analyzed them together in a 1:1 mix (10, 50, 100, 500, and 1,000 ng) to correct for any competition for ionization and suppression effects that may have been taking place. It is more sensible to use the peak properties derived from the mix analysis, as this is closer to the real sample that was analyzed. We preferred to use the peak areas rather than the peak heights, because the latter tend to plateau as the amount of peptide injected increases, due to peak broadening. Using the areas under the peak takes into consideration the effect of peak broadening and is therefore a better property to use. When present at a 1:1 ratio, at increasing peptide concentrations there is a positive correlation between the peak areas (calculated from the extracted-ion chromatograms [XICs]) generated using the *m/z* values of the standard peptides with a mass tolerance of 5 ppm) corresponding to the nonhydroxylated standard and the hydroxylated standard, as follows.

(1) $$\text{Peak}\;\text{area}{\;}_{\left(\text{hydroxylated}\;\text{peptide}\right)}=0.647\times \text{peak}\;{\text{area}}_{\left(\text{nonhydroxylated}\;\text{peptide}\right)}$$

Using the equation above, we are able to measure the stoichiometry of hydroxylation, from previously acquired data, as follows.

The peak area measured (MA) for the nonhydroxylated peptide was

$$\text{MA}{\;}_{\left(\text{nonhydroxylated}\right)}=4,731,674.$$

The peak area measured from the XIC of the hydroxylated peptide was

$$\text{MA}{\;}_{\left(\text{hydroxylated}\right)}=309,968.$$

After correcting the peak area for the hydroxylated peptide using Equation 1, we obtained a corrected peak area ${\text{MA}}_{\left(\text{hydroxylated}\right)}^{\prime }$, where ${\text{MA}}_{\left(\text{hydroxylated}\right)}^{\prime }={\text{MA}}_{\left(\text{hydroxylated}\right)}/0.647$,

$${\text{MA}}_{\left(\text{hydroxylated}\right)}^{\prime }=479,085.0077.$$

Finally, we were able to calculate the relative amounts of hydroxylated peptide and nonhydroxylated peptide using the ${\text{MA}}_{\left(\text{hydroxylated}\right)}^{\prime }$ and the ${\text{MA}}_{\left(\text{nonhydroxylated}\right)}$:

$$\text{Ratio}\left(\frac{\text{hydroxylated}}{\text{nonhydroxylated}}\right)=\frac{{\text{MA}}^{\prime }}{\text{MA}}$$

$$\text{Ratio}\left(\frac{\text{hydroxylated}}{\text{nonhydroxylated}}\right)=\frac{479,085.0077}{4,731,674}$$

$$\text{Ratio}\left(\frac{\text{hydroxylated}}{\text{nonhydroxylated}}\right)=0.101$$

$$\text{Ratio}\left(\frac{\text{hydroxylated}}{\text{nonhydroxylated}}\right)=10.1\%.$$

### Cells and Cell Culture

HeLa cells and U2OS cells were grown in DMEM supplemented with 10% FBS and antibiotics. Stable cell lines were created using the manufacturer’s instructions. These cells were maintained in 10% FBS/DMEM supplemented with 200 μg/ml hygromycin or 500 μg/ml G418. hTERT-RPE1 cells were grown in DMEM:F12 medium supplemented with 10% FBS and antibiotics.

### Antibodies and Antibody Dilutions

The following antibodies were used: anti-PHD1 (Bethyl A300-326A; Novus NBP1-40773), anti-PHD2 (Bethyl A300-322A), anti-PHD3 (Bethyl A300-327A), anti-Cep192 1:200 (immunofluorescence) and 1:1,000 (western) (Bethyl A302-324A; Novus NBP-84634), anti-actin (Cell Signaling 3700), anti-tubulin 1:400 (immunofluorescence) and 1:1,000 (western) (Sigma T9026; AbD Serotec MCA77G), anti-cyclin B (Cell Signaling 4138), anti-pericentrin 1:1,000 and 1:500 (Abcam ab4448, ab28144), anti-γ-tubulin 1:500 (Abcam ab11317; Sigma T5326), anti-acetylated tubulin 1:200 (Sigma T7451), anti-centrin 1:1,000 (Millipore 04-1624), anti-HIF-1α (BD Biosciences 610958), anti-Skp2 (Cell Signaling 4358; Santa Cruz sc-7164), anti-Glut3 (AnaSpec 53520), and anti-ubiquitin (Santa Cruz sc-8017). Secondary antibodies linked to HRP were purchased from Cell Signaling (rabbit) and Sigma (mouse). Fluorescence-labeled secondary antibodies were obtained from Jackson Laboratories and used at a dilution of 1:150.

### Flow Cytometric Analysis of Cell-Cycle Distribution

Adherent and detached cells were harvested, pooled, washed once in PBS, and fixed in ice-cold 70% (v/v) ethanol in distilled water. Cells were then washed twice in PBS and resuspended in Guava Cell Cycle reagent (Millipore). After incubation at room temperature for 30 min, cells were analyzed for cell-cycle distribution with a Guava easyCyte HT machine and software. Red fluorescence (585 ± 42 nm) was evaluated on a linear scale, and pulse-width analysis was used to exclude cell doublets and aggregates. Cells with a DNA content between 2N and 4N were designated as being in the G1, S, or G2/M phase of the cell cycle. The number of cells in each compartment of the cell cycle was expressed as a percentage of the total number of cells present.

### Immunoblotting and Immunoprecipitation

Whole-cell extracts were prepared by cell lysis in 20 mM Tris (pH 7.5), 150 mM NaCl, 0.5% Triton X-100, and EDTA-free complete protease inhibitor mix (Roche), resolved by SDS-PAGE, transferred to PVDF membranes, and probed with the indicated antibodies. For immunoprecipitation, cells were lysed and antibody was added to cleared lysates for 1.5 hr, followed by a 1.5 hr rotation with protein G Sepharose beads (Pierce) at 4°C. The beads were then washed three times with PBS buffer. The proteins bound to the beads were dissolved in SDS sample buffer, separated by SDS-PAGE, and blotted with the indicated antibodies. For ubiquitination assays, cells were lysed in 1% SDS buffer and boiled for 30 min prior to 10-fold dilution with the standard lysis buffer mentioned above. Lysates were used to immunoprecipitate ubiquitin overnight, and samples were processed as above.

### Immunofluorescence Microscopy

Cells were fixed, permeabilized, and blocked as previously described (Posch et al., 2010). A DeltaVision RT microscope equipped with a 100× objective was used for image acquisition. Images were analyzed using OMERO (Open Microscopy Environment) and custom-made built-in tools using MATLAB (MathWorks) made by M. Porter (code available on request).

### Hypoxia Inductions and MG132 Treatment

Cells were incubated at 1% O_2_ in an Invivo 300 hypoxia workstation (Ruskinn). For hypoxia-mimetic conditions, cells were treated with 100 μM desferrioxamine. MG132 (Merck Biosciences) was added 4 hr prior to cell harvesting.

### LC-MS Analysis

The digests were analyzed using nano-LC (RSLC; Thermo Scientific) coupled to Q Exactive (Thermo Scientific). The peptides were loaded in 5% formic acid and resolved on a 50 cm reverse-phaseC18 column (Thermo Scientific) using a multistep gradient of acetonitrile (5%–60% acetonitrile). The peptides eluted directly into the mass spectrometer’s sampling region, and the spray was initiated by applying 1.2 kV to the fused silica emitter (New Objective). The data were acquired under the control of Xcalibur software (Thermo Scientific) in a data-dependent mode selecting the 15 most intense ions for sequencing in tandem MS. In addition to the dynamic selection of the 15 most intense ions, the peptide ions corresponding to the peptides WHLSSLAPPYVK and WHLSSLAPPYVKGVDESGDVFR, hydroxylated and nonhydroxylated, were added to an inclusion list for fragmentation regardless of their intensity. For targeted MS analysis, the ions corresponding to the hydroxylated and nonhydroxylated peptides WHLSSLAPPYVK and WHLSSLAPPYVKGVDESGDVFR were exclusively selected for tandem MS. The peptides were sequenced manually.

## Supplemental Information

Document S1. Supplemental Experimental Procedures and Figures S1–S6
