# Supplementary material for: PHD1 Links Cell-Cycle Progression to Oxygen Sensing through Hydroxylation of the Centrosomal Protein Cep192
Source: Dev Cell. 2013 Aug 26;26(4):381–92. doi: 10.1016/j.devcel.2013.06.014 (PMC3757158; doi:10.1016/j.devcel.2013.06.014)
Supplement: Document S1. Supplemental Experimental Procedures and Figures S1–S6 [file mmc1.pdf]

Developmental Cell, Volume *26*

Supplemental Information

PHD1 Links Cell-Cycle Progression to Oxygen Sensing through Hydroxylation of the Centrosomal Protein Cep192

Sandra C. Moser, Dalila Bensaddek, Brian Ortmann, Jean-Francois Maure, Sharon Mudie, J. Julian Blow, Angus I. Lamond, Jason R. Swedlow, and Sonia Rocha

Supplementary Information Inventory

Supplementary Figures and Figure legends

The six Supplemental Figures are related to main Figures 1-5 as follows:

- Figure S1, related to Figure 1: Validation of siRNA efficiency and PHD1 depleted cells display a prometaphase arrest
- Figure S2, related to Figure 2: PHD1 is required for the centrosomal localization of Cep192
- Figure S3, related to Figure 3: PHD1 colocalizes with Cep192 at centrosomes in mitosis
- Figure S4, related to Figure 3: Synthetic standard of hydroxylated Cep192 and Fragment ions incorporating the hydroxylation in Cep192.
- Figure S5, related to Figure 5: Cep192 mRNA and protein levels are independent of HIF
- Figure S6, related to Figure 5: Differential effects of hypoxia and DFX treatment to centrosomal Cep192.

Supplementary experimental procedures

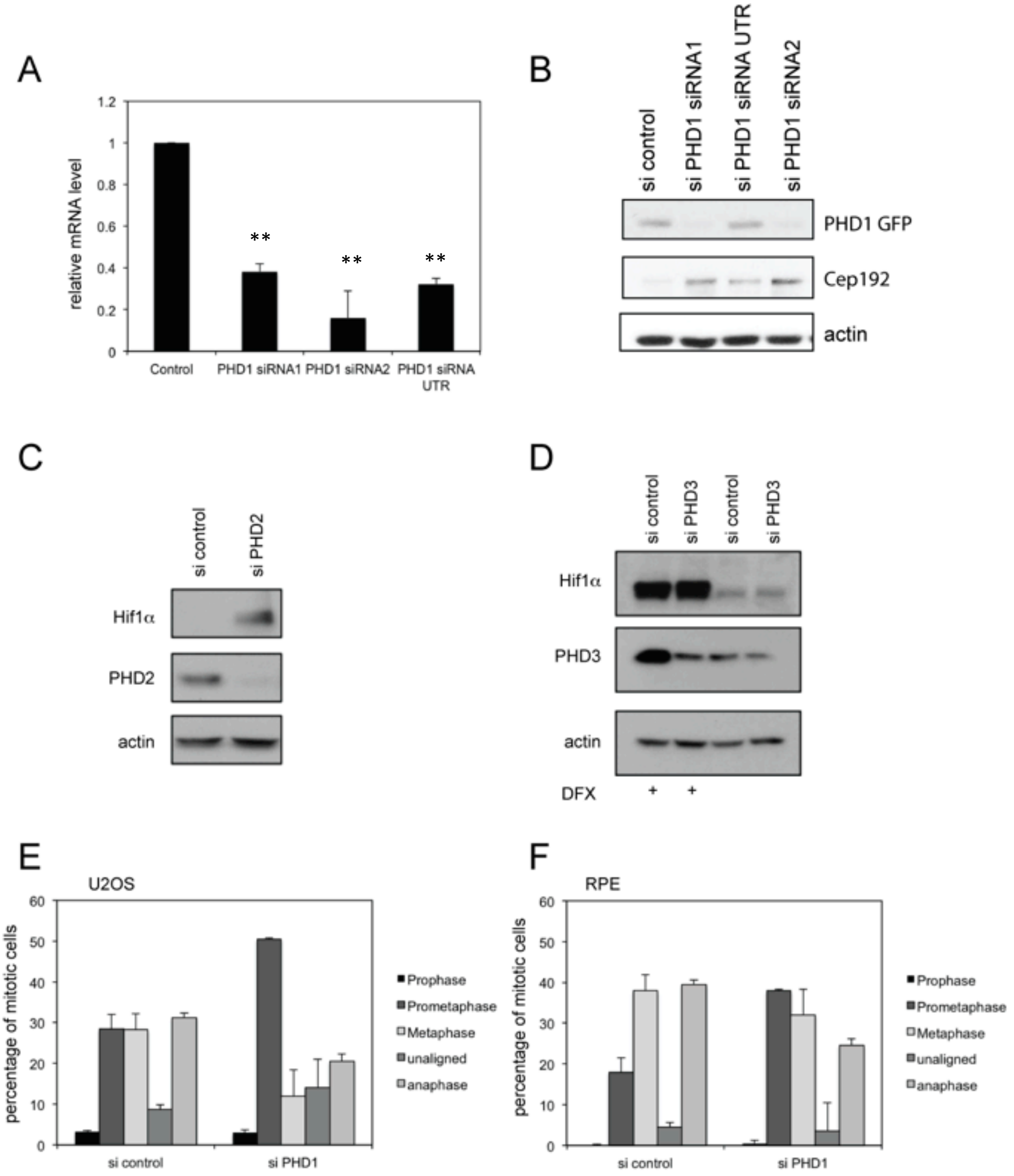
A
relative mRNA level
1.2
1
0.8
0.6
0.4
0.2
0
**
Control
PHD1 siRNA1
PHD1 siRNA2
PHD1 siRNA UTR
B
si control
si PHD1 siRNA1
si PHD1 siRNA UTR
si PHD1 siRNA2
PHD1 GFP
Cep192
actin
C
si control
si PHD2
Hif1α
PHD2
actin
D
si control
si PHD3
Hif1α
PHD3
actin
DFX
+
E
U2OS
percentage of mitotic cells
60
50
40
30
20
10
0
si control
si PHD1
Prophase
Prometaphase
Metaphase
unaligned
anaphase
F
RPE

Fig. S1

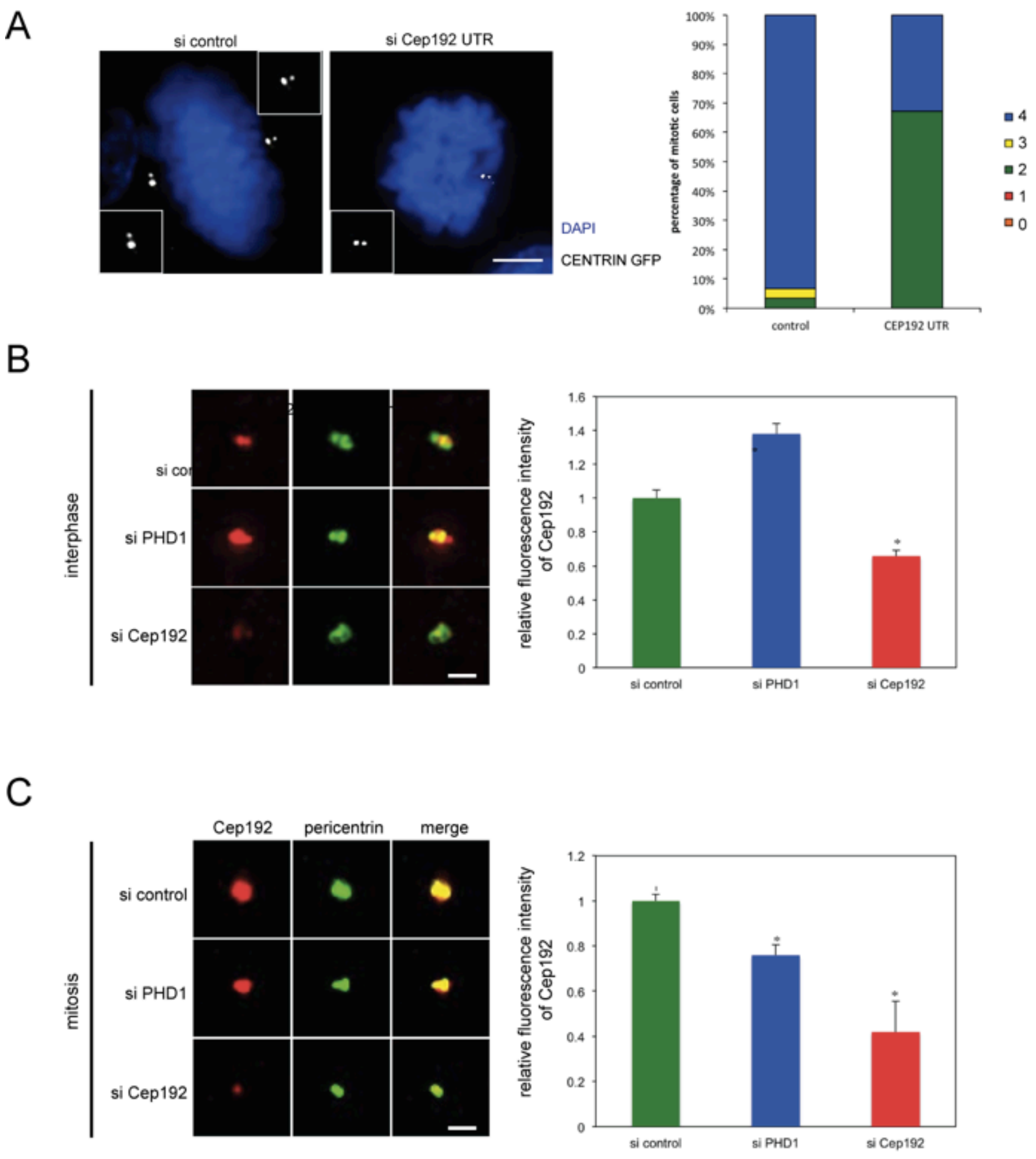
A
si control
si Cep192 UTR
DAPI
CENTRIN GFP
percentage of mitotic cells
100%
90%
80%
70%
60%
50%
40%
30%
20%
10%
0%
control
CEP192 UTR
4
3
2
1
0
B
interphase
si PHD1
si Cep192
relative fluorescence intensity of Cep192
1.6
1.4
1.2
1
0.8
0.6
0.4
0.2
0
si control
si PHD1
si Cep192
C
Cep192
pericentrin
merge
mitosis
si control
si PHD1
si Cep192
relative fluorescence intensity of Cep192
1.2
1
0.8
0.6
0.4
0.2
0
si control
si PHD1
si Cep192

Fig. S2

A

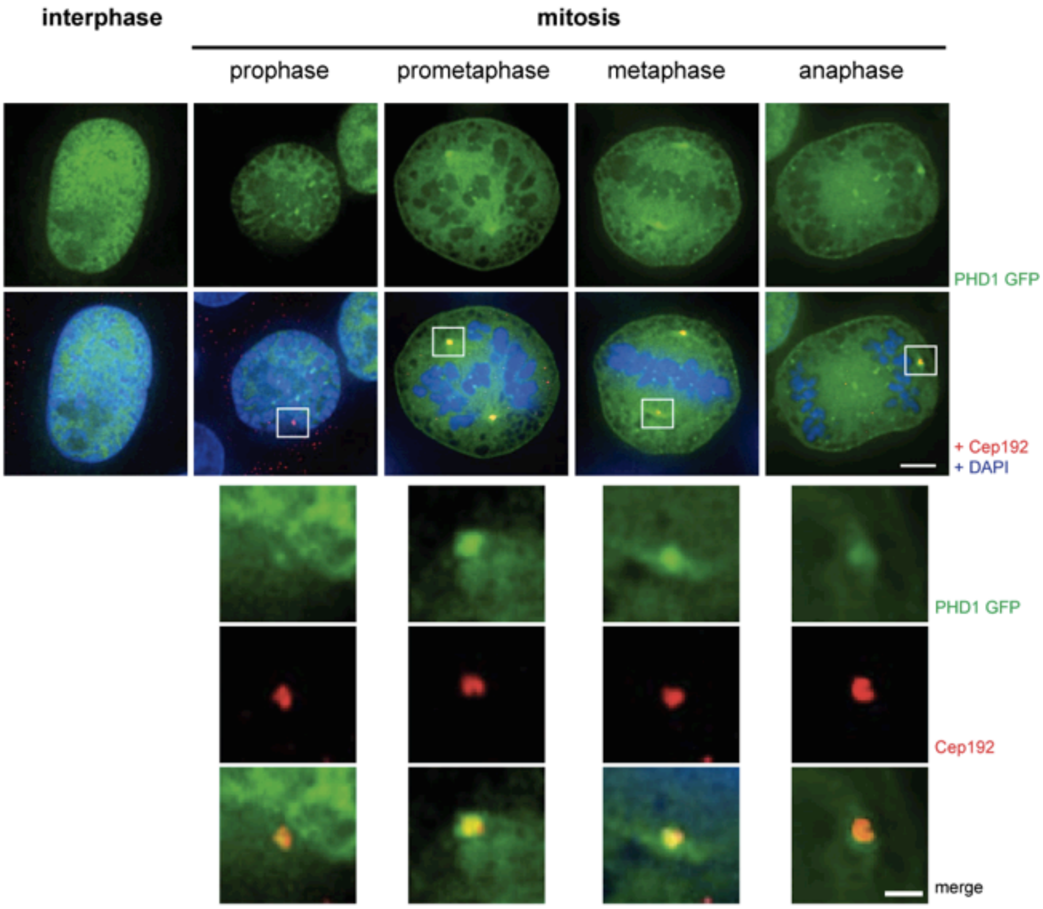

interphase
mitosis
prophase
prometaphase
metaphase
anaphase
PHD1 GFP
+ Cep192
+ DAPI
PHD1 GFP
Cep192
merge

B

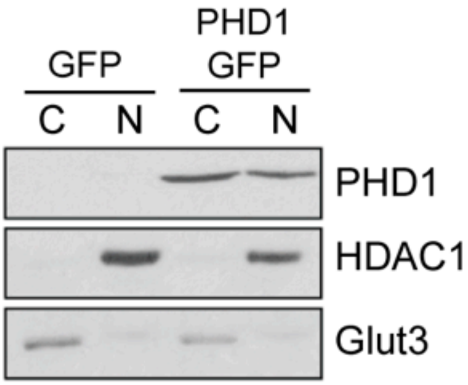

GFP
PHD1 GFP
C
N
C
N
PHD1
HDAC1
Glut3

Fig. S3

A

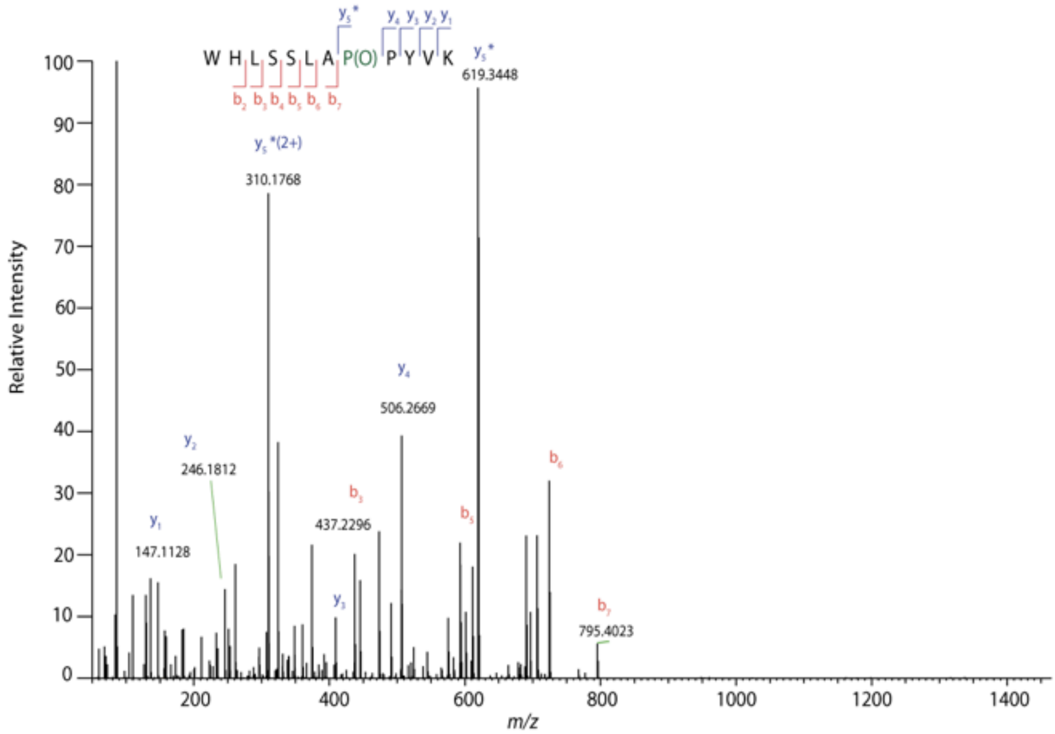

B

b	b^{+2}				y	y^{+2}
---	---	1	W	12	---	---
324.1455#	162.5764	2	H	11	1227.6732	614.3366
437.2296*#	219.1184	3	L	10	1090.6143	545.8072
524.2616*	262.6344	4	S	9	977.5302	489.2651
611.2936*	306.1504	5	S	8	890.4982	445.7491
724.3777*	362.6925	6	L	7	803.4662	402.2331
795.4148*	398.211	7	A	6	690.3821#	345.6911
908.4625	454.7313	8	P (OH)	5	619.3450*#	310.1725*
1005.5152	503.2576	9	P	4	506.2973*#	253.6523#
1168.5786	584.7893	10	Y	3	409.2445*#	205.1259#
1267.6470	634.3235	11	V	2	246.1812*#	123.5942
---	---	12	K	1	147.1128*	74.06

Fig.S4

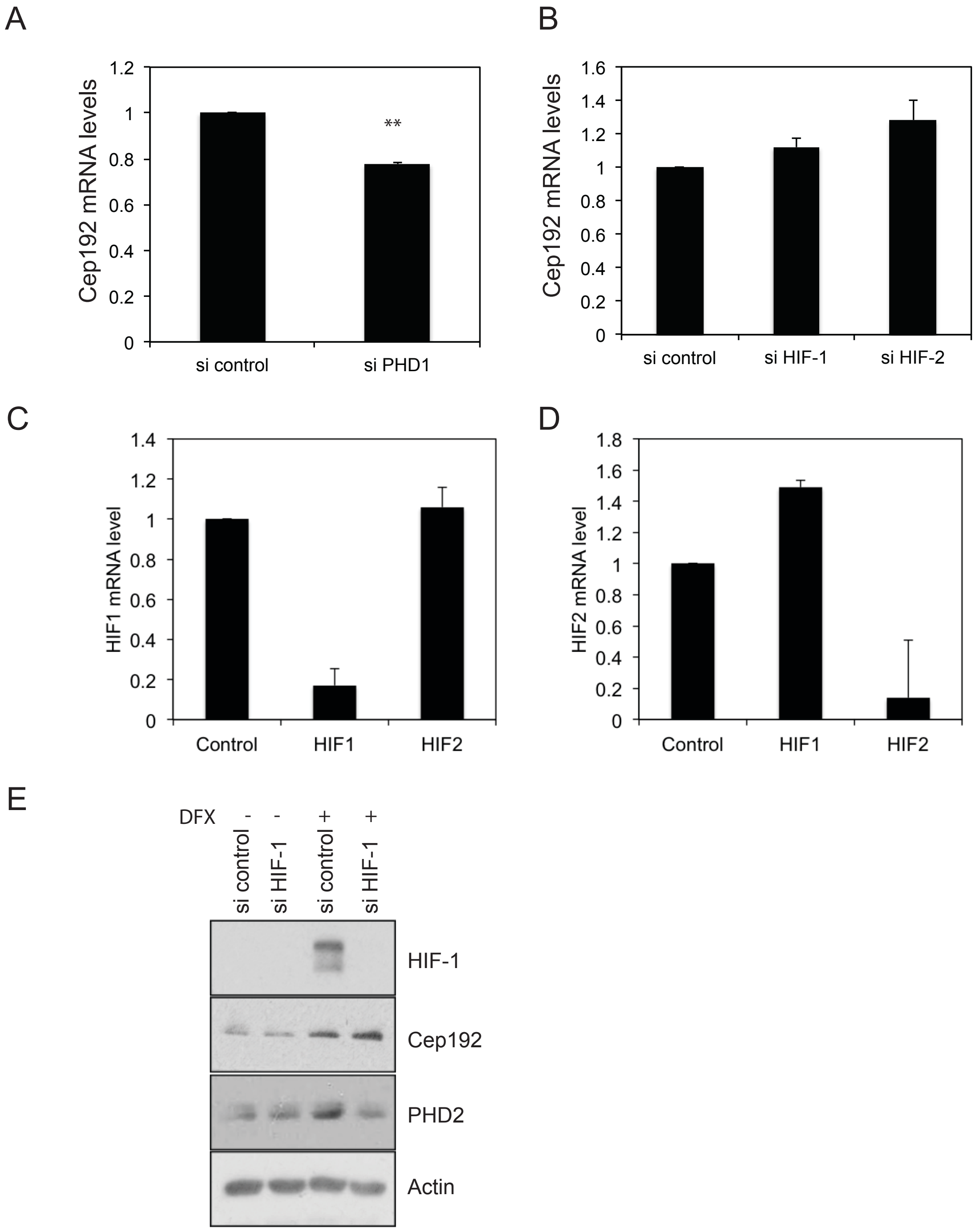
A
Cep192 mRNA levels
1.2
1
0.8
0.6
0.4
0.2
0
**
si control
si PHD1
B
Cep192 mRNA levels
1.6
1.4
1.2
1
0.8
0.6
0.4
0.2
0
si control
si HIF-1
si HIF-2
C
HIF1 mRNA level
1.4
1.2
1
0.8
0.6
0.4
0.2
0
Control
HIF1
HIF2
D
HIF2 mRNA level
1.8
1.6
1.4
1.2
1
0.8
0.6
0.4
0.2
0
Control
HIF1
HIF2
E
DFX - - + +
si control
si HIF-1
si control
si HIF-1
HIF-1
Cep192
PHD2
Actin

Fig. S5

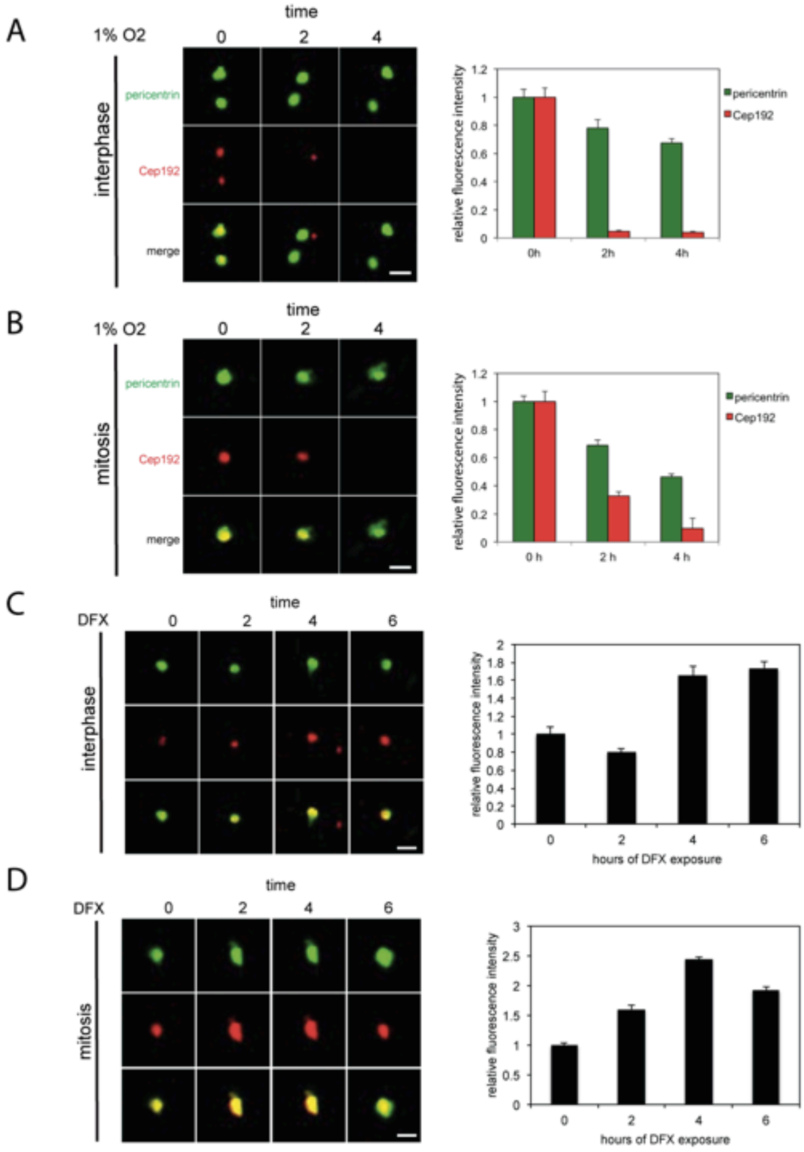
A
1% O2
time
0
2
4
interphase
pericentrin
Cep192
merge
relative fluorescence intensity
pericentrin
Cep192
0h
2h
4h
B
1% O2
time
mitosis
pericentrin
Cep192
merge
relative fluorescence intensity
0 h
2 h
4 h
C
DFX
time
0
2
4
6
interphase
relative fluorescence intensity
hours of DFX exposure
D
DFX
time
mitosis
relative fluorescence intensity
hours of DFX exposure

Figure S6

Supplemental Figure Legends:

Figure S1, related to Figure 1. Validation of siRNA knockdown efficiency. (A) U2OS cells were treated with siRNAs targeting PHD1 for 48h. mRNA abundance of PHD1 was determined by real time PCR. Bars represent average of three experiments, error bars indicate ± 1 s.d. p value significant according to student's test $**p<0.01$. (B) Extracts from U2OS PHD1-GFP cells treated with the indicated siRNAs were blotted with the indicated antibodies. (C) Extracts from HeLa cells treated with the indicated siRNAs were blotted with the indicated antibodies. (D) Extracts from HeLa cells treated with the indicated siRNAs +/- DFX were blotted with the indicated antibodies. (E-F) PHD1 depleted cells display a prometaphase arrest. (E) U2OS cells and (F) RPE cells were treated with PHD1 siRNA and the mitotic distribution was determined. Bars represent percentage of cells in a particular mitotic stage (average of two independent experiments, error bars indicate ± 1s.d.).

Figure S2, related to Figure 2. PHD1 is required for the centrosomal localization of Cep192. (A) Cep192 is required for centriole duplication. To confirm the specificity of the centriole duplication phenotype HeLa Kyoto cells expressing centrin GFP were depleted of Cep192 with a different set of siRNA targeting Cep192 (Cep192UTR) and stained with DAPI (blue). Scale bar 5μm. Inlays focus on centrioles. Centriole numbers in control and Cep192 depleted cells were quantified (right) n=50. (B-C) RPE cells were depleted of PHD1 or Cep192 and stained for Cep192 (red) and pericentrin (green). Figures display centrosomes in interphase (top left) and mitosis (bottom left). Scale bar 1 μm. The relative fluorescence intensity of Cep192 signal in interphase (top right) and mitosis (bottom right) was determined (n=20, error bars indicate ± 1 s.e.m, p value significant according to student's test $*p<0.05$).

Figure S3, related to Figure 3. PHD1 colocalizes with Cep192 at centrosomes in mitosis

(A) Immunofluorescence images of HeLa cells expressing GFP PHD1 stained for Cep192 (red) and DNA (blue). Scale bar 5 μm. Inlays focus on the centrioles. Scale bar 1 μm. (B) Cells expressing PHD1 GFP or GFP alone were subjected to

subcellular fractionation. C representing the cytoplasmic and N representing the nuclear fraction. Fractions were blotted with the indicated antibodies.

Figure S4, related to Figure 3. (A) The product ion spectrum of the synthetic standard corresponding to Cep192 hydroxylated peptide. MS-MS spectrum of the doubly charged ion at *m/z* 707.3797 showing b and y fragment ions typical for HCD fragmentation allowing the near complete sequencing of the peptide as WHLSSLAP(OH)PYVK and the determination of the site of hydroxylation. (B) Fragment ions incorporating the hydroxylation in Cep192. Theoretical fragment ions formed upon collisional activation (HCD) of the hydroxylated peptide WHLSSLAP(OH)PYVK, highlighted in purple are the fragment ions incorporating the modification and thus the mass increment of 15.999Da. * denotes the fragment ions observed in the tandem MS spectrum of the hydroxylated synthetic standard, # denotes fragment ions observed in the tandem MS spectrum of the endogenous hydroxylated peptide.

Figure S5, related to Figure 5. Cep192 mRNA and protein levels are independent of HIF. U2OS cells were treated with siRNAs targeting PHD1 (A) and HIF1 and HIF2 (B) for 48h. mRNA abundance of Cep192 was determined by real time PCR. Bars represent average of three experiments, error bars indicate ± 1 s.d. p value significant according to student's test $^{**}p<0.01$. mRNA levels of HIF1 (C) and HIF2 (D) showing the efficiency of siRNA knockdown. Bars represent average of three experiments, error bars indicate ± 1 s.d. (E) Extracts from U2OS depleted of HIF1 and treated or not with DFX were blotted with the indicated antibodies.

Figure S6, related to Figure 5. Differential effects of hypoxia and DFX treatment to centrosomal Cep192. (A) Hypoxia leads to a decrease of Cep192 and pericentrin levels at the centrosome in interphase (A) and mitosis (B). HeLa cells were exposed to 1% O_2 for the indicated timepoints. Cells were fixed and stained for Cep192 (red) and pericentrin (green). Quantification of relative fluorescence signals is shown in panels on the right. Exposure of cells to DFX increases Cep192 levels at the centrosome in interphase (C) and mitosis (D). Cells were treated with DFX for the indicated timepoints. Cells were fixed and stained for Cep192 (red) and pericentrin

(green). Scale bar 1 μm. Quantification of relative fluorescence signals is shown in panels on the right. Error bars indicate ± 1 s.e.m.

Supplementary experimental procedures

Additional siRNA sequences

PHD1_B-GCUGCAUCACCUGUAUCUA

PHD1_C-ACAGAAAGGUGUCCAAGUA

HIF-2α – CAGCAUCUUUGACAGUTT {Culver et al, 2011}

RT-qPCR and primers sequences

Quantitative RT-PCR was performed using cDNA templates (cDNA synthesis was performed using Quantitect Reverse Transcription kit (Qiagen)) amplified using specific primer sets and the Stratagene Brilliant II SYBR green qPCR mix according to the manufacturer instructions. Amplification and detection were performed using a Stratagene Mx3005P detection system. Sample values obtained with specific primer sets were normalized to β-actin primer set values.

PCR primer sequences:
Actin
For-CTGGGAGTGGGTGGAGGC
Rev-TCAACTGGTCTCAAGTCAGTG

PHD1
For-CTGGGCAGCTATGTCATCAA
Rev-AAATGAGCAACCGGTCAAAG

HIF-1α

For-CACTGAGGCAGTGGAGACAG

Rev-TGCAGTCCCAGCTACTTGTG

HIF-2α

For-TTTGATGTGGAAACGGATGA

Rev-GGAACCTGCTCTTGCTGTTC

Cep192

For-TGCTTGTTCCTCCATATCCA

Rev-CGATTATCTTGCATGTTGGCT
